# Improved control configuration of PWM rectifiers based on neuro-fuzzy controller

**DOI:** 10.1186/s40064-016-2781-5

**Published:** 2016-07-21

**Authors:** Hakan Acikgoz, O. Fatih Kececioglu, Ahmet Gani, Ceyhun Yildiz, Mustafa Sekkeli

**Affiliations:** Department of Electrical Science, Kilis 7 Aralik University, 79000 Kilis, Turkey; Department of Electrical and Electronics, Faculty of Engineering, Kahramanmaras Sutcu Imam University, Kahramanmaras, Turkey

**Keywords:** PWM rectifiers, Neuro-fuzzy controller, Total harmonic distortion, Power quality

## Abstract

It is well-known that rectifiers are used widely in many applications required AC/DC transformation. With technological advances, many studies are performed for AC/DC converters and many control methods are proposed in order to improve the performance of these rectifiers in recent years. Pulse width modulation (PWM) based rectifiers are one of the most popular rectifier types. PWM rectifiers have lower input current harmonics and higher power factor compared to classical diode and thyristor rectifiers. In this study, neuro-fuzzy controller (NFC) which has robust, nonlinear structure and do not require the mathematical model of the system to be controlled has been proposed for PWM rectifiers. Three NFCs are used in control scheme of proposed PWM rectifier in order to control the dq-axis currents and DC voltage of PWM rectifier. Moreover, simulation studies are carried out to demonstrate the performance of the proposed control scheme at MATLAB/Simulink environment in terms of rise time, settling time, overshoot, power factor, total harmonic distortion and power quality.

## Background

With advances in power electronics and microprocessors, power electronics technology has widely used for many applications. Nowadays, AC/DC converters, which are also known as rectifiers, are used in adjustable speed drives, uninterruptible power supply systems, photovoltaic systems, battery energy storage systems, DC motor drives and communication systems (Singh et al. 2004; Blasko and Kaura 1997). AC to DC conversion has been performed by an uncontrollable diode rectifier or a phase controlled thyristor rectifier. Although these rectifiers have high reliability, simple structure and low cost, they have many disadvantages such as low power factor, high THD and unidirectional power flow. Moreover, these rectifiers that produce high harmonic currents are actually a harmonic source and have caused harmonic problems (Dannehl et al. 2009; Sekkeli et al. 2015). To solve these problems, new standards have been introduction by a number of countries and international organizations to limit harmonics formed in the current drawn from main supply by rectifiers. During the past 20 years, the interest in AC/DC rectifiers has been growing by day by due to the increasing concern about the harmonic pollution in the power systems. Thanks to the rapid development of technology, new rectifier type for three-phase AC/DC conversation have been developed such as PWM rectifier (Wu et al. 1991). PWM rectifiers used for AC-DC conversion have many advantages like controllable DC voltage, fast dynamic response, controllable reactive power, unity power factor, low harmonic distortion and bidirectional power flow (Bouafia et al. 2010a, b). Generally, the control techniques of PWM rectifiers can be classified into two types: Voltage oriented control (VOC) and direct power control (DPC). VOC based on internal current control loops became very popular method. Another control method is called as DPC which has not internal current loops and PWM blocks (Malinowski et al. 2001; Malinowski and Kazmierkowski 2003; Monfared et al. 2010). The main goal of these control techniques is to eliminate the current harmonics and to regulate the DC bus voltage. In the control of PWM based rectifiers, DC bus voltage and dq-axis currents are generally controlled by proportional-integral (PI) controllers due to their simple structure. PI controllers need linear mathematical model of system. Moreover, it is known that PI controllers have many disadvantages such as slow response, large overshoots and oscillations (Cortes et al. 2008; Blasko and Kaura 1997). To cope with these problems, many control methods have been proposed by many academics and researchers, namely fuzzy logic controllers (FLC), robust H∞ controller, linear quadratic regulator (LQR), sliding mode control (SMC) and predictive control (PC). These intelligent controllers have used for many industrial applications and rectifier systems to obtain a good performance in both transient and steady state from PWM rectifier (Antoniewicz and Kazmierkowski 2006; Yu et al. 2010; Zhao et al. 2011; Jiabing et al. 2011; Bouafia and Krim 2008; Djerioui et al. 2014). NFC that has nonlinear, robust structure and based on FLC whose functions are realized by ANN is one of these intelligent controllers (Zadeh 1965; Jang et al. 1997; Mohagheghi et al. 2007). In this paper, the robust and nonlinear control strategy based NFC controllers are proposed for DC bus voltage and dq-axis currents control of PWM rectifier in order to achieve a good dynamic response. NFC controllers designed for DC voltage and dq-axis currents have two inputs, single output and six layers. This paper is organized as follows: Power circuit and mathematical model of PWM rectifier is given in first section. The determination of electrical parameters in PWM rectifier and design of PI controller are presented in second section. The description of the NFC and its training algorithm are explained in third section. The simulation results related to proposed controller are comprehensively presented in fourth section. The final section provides the conclusions of this study.

## Mathematical model of PWM rectifier

The three-phase PWM rectifiers are widely used in a wide diversity of applications in recent years. These rectifiers have many advantages such as bi-directional power flow, low harmonic distortion of line current, unity power factor, control of DC bus voltage (Blasko and Kaura 1997; Kazmierkowski et al. 2002).

**Fig. 1:**
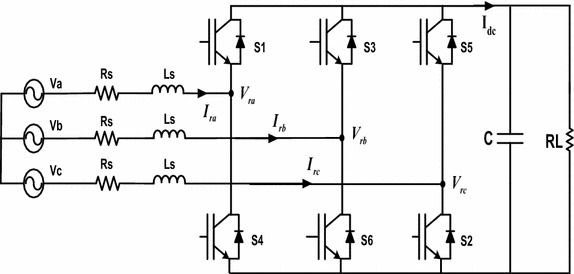
Block diagram of three-phase PWM rectifier

The structure of three-phase PWM rectifier is as shown in Fig. 1. The source phase voltages are expressed as:

1 $${\text{V}}_{\text{a}} = {\text{V}}_{\text{m}} \sin\uptheta$$

2 $${\text{V}}_{\text{b}} = {\text{V}}_{\text{m}} \sin (\uptheta - 2\uppi/3)$$

3 $${\text{V}}_{\text{c}} = {\text{V}}_{\text{m}} \sin (\uptheta + 2\uppi/3)$$

The mathematical model of PWM rectifier in abc frame can be expressed as: (Blasko and Kaura 1997).

4 $$\left[ {\begin{array}{*{20}c} {{\text{L}}_{\text{s}} \frac{{{\text{dI}}_{\text{ra}} }}{\text{dt}}} \\ {{\text{L}}_{\text{s}} \frac{{{\text{dI}}_{\text{rb}} }}{\text{dt}}} \\ {{\text{L}}_{\text{s}} \frac{{{\text{dI}}_{\text{rc}} }}{\text{dt}}} \\ {{\text{L}}_{\text{s}} \frac{{{\text{du}}_{\text{c}} }}{\text{dt}}} \\ \end{array} } \right] = \left[ {\begin{array}{*{20}c} { - {\text{R}}_{\text{s}} } & 0 & 0 & 0 \\ 0 & { - {\text{R}}_{\text{s}} } & 0 & 0 \\ 0 & 0 & { - {\text{R}}_{\text{s}} } & 0 \\ {{\text{S}}_{\text{a}} } & {{\text{S}}_{\text{b}} } & {{\text{S}}_{\text{c}} } & { - 1} \\ \end{array} } \right]\left[ {\begin{array}{*{20}c} {{\text{I}}_{\text{ra}} } \\ {{\text{I}}_{\text{rb}} } \\ {{\text{I}}_{\text{rc}} } \\ {{\text{I}}_{\text{L}} } \\ \end{array} } \right] + \left[ {\begin{array}{*{20}c} {{\text{V}}_{\text{a}} - {\text{V}}_{\text{ra}} } \\ {{\text{V}}_{\text{b}} - {\text{V}}_{\text{rb}} } \\ {{\text{V}}_{\text{c}} - {\text{V}}_{\text{rc}} } \\ 0 \\ \end{array} } \right]$$

5 $$\left[ {\begin{array}{*{20}c} {{\text{V}}_{\text{ra}} } \\ {{\text{V}}_{\text{rb}} } \\ {{\text{V}}_{\text{rc}} } \\ \end{array} } \right] = \left[ {\begin{array}{*{20}c} {2/3} & { - 1/3} & { - 1/3} \\ { - 1/3} & {2/3} & { - 1/3} \\ { - 1/3} & { - 1/3} & {2/3} \\ \end{array} } \right]\left[ {\begin{array}{*{20}c} {S_{a} } \\ {S_{b} } \\ {S_{c} } \\ \end{array} } \right]V_{dc}$$

where, L_s_ and R_s_ are grid inductance and resistance, respectively. I_a_, I_b_ and I_c_ are grid phase currents; V_ra_, V_rb_ and V_rc_ are the rectifier input voltages. V_ra_, V_rb_ and V_rc_ voltages can be found by opening and closing in accordance with the switching elements in the structure of rectifier to obtain the DC link voltage. Where, S_a_, S_b_ and S_c_ show switching functions. These functions get 0, if the switch is off; if it is on, then they are 1. Clarke’s matrix in α–β frame can be described as following (Blasko and Kaura 1997):

6 $${\text{T}} = \left[ {\begin{array}{*{20}c} 1 & { - 1/2} & { - 1/2} \\ 0 & {\sqrt 3 /2} & { - \sqrt 3 /2} \\ \end{array} } \right]$$

According to Clake’s transformation, the dynamic model of PWM rectifier can be defined as:

7 $$\left[ {\begin{array}{*{20}c} {{\text{V}}_{\upalpha } } \\ {{\text{V}}_{\upbeta } } \\ \end{array} } \right] = {\text{L}}_{\text{s}} \left[ {\begin{array}{*{20}c} {\frac{{{\text{dI}}_{\upalpha } }}{\text{dt}}} \\ {\frac{{{\text{dI}}_{\upbeta } }}{\text{dt}}} \\ \end{array} } \right] + \left[ {\begin{array}{*{20}c} {{\text{R}}_{\text{s}} } & 0 \\ 0 & {{\text{R}}_{\text{s}} } \\ \end{array} } \right]\left[ {\begin{array}{*{20}c} {{\text{I}}_{\upalpha } } \\ {{\text{I}}_{\upbeta } } \\ \end{array} } \right] + \left[ {\begin{array}{*{20}c} {{\text{V}}_{{{\text{r}}\upalpha }} } \\ {{\text{V}}_{{{\text{r}}\upbeta }} } \\ \end{array} } \right]$$

If Park’s transformation is applied to rectifier system then following equation can be derived:

8 $$\left[ {\begin{array}{*{20}c} {{\text{V}}_{\text{d}} } \\ {{\text{V}}_{\text{q}} } \\ \end{array} } \right] = {\text{L}}_{\text{s}} \left[ {\begin{array}{*{20}c} {\frac{{{\text{dI}}_{\text{d}} }}{\text{dt}}} \\ {\frac{{{\text{dI}}_{\text{q}} }}{\text{dt}}} \\ \end{array} } \right] + \left[ {\begin{array}{*{20}c} {{\text{R}}_{\text{s}} } & { - \upomega {\text{L}}_{\text{s}} } \\ {\upomega {\text{L}}_{\text{s}} } & {{\text{R}}_{\text{s}} } \\ \end{array} } \right]\left[ {\begin{array}{*{20}c} {{\text{I}}_{\text{d}} } \\ {{\text{I}}_{\text{q}} } \\ \end{array} } \right] + \left[ {\begin{array}{*{20}c} {{\text{V}}_{\text{rd}} } \\ {{\text{V}}_{\text{rq}} } \\ \end{array} } \right]$$

Angle value (θ) required for above transformations can be found with PLL (phase locked loop) in MATLAB/Simulink or it can be obtain by using abc–αβ transformation.

## Determination of electrical parameters used in PWM rectifier and design of PI controller

Input inductor must be designed very carefully in order to obtain good performance from rectifier. Low inductance value causes the increase in current ripple and the performance of rectifier depends on impedance of the grid. The high inductance value reduces current ripple but it limits the operating range of the rectifier (Wang and Yin 2008). Consequently, the maximum inductance value can be determined as follows:

9 $$\frac{{(2{\text{U}}_{\text{dc}} - 3{\text{U}}_{\text{m}} ){\text{U}}_{\text{m}} {\text{T}}_{s} }}{{2{\text{U}}_{\text{dc}} \Delta {\text{I}}_{ \hbox{max} } }} \le {\text{L}} \le \frac{{2{\text{U}}_{\text{dc}} }}{{3{\text{I}}_{\text{m}} \upomega }}$$

where, U_m_ is the phase voltage amplitude of the grid, T_s_ is the switching period, I_m_ is the amplitude of the grid current, ω is the angular frequency, and ∆I_max_ is the allowed maximal ripple of the grid current. The determination of the value of C is very important because C has key role in fixed the DC voltage. Also, the value of C should be as small as possible in order to provide fast tracking of the reference DC voltage. The value of C can be found as the following equations:

10 $${\text{C}} \le \frac{{{\text{t}}_{\text{r}}^{*} }}{{{\text{R}}_{\text{L}} \ln \left( {6 - \frac{{8.27{\text{U}}_{\text{m}} }}{{{\text{U}}_{\text{dc}} }}} \right)}}$$

11 $${\text{C}} > \frac{1}{{2{\text{R}}_{\text{L}} \Delta {\text{U}}_{\text{dc}}^{*} }}$$

where, ∆U_dc_ = (U_dc_ − U_dcmin_)/U_dc_, t_r_^*^ is the rising time for the output voltage and R_L_ is the output load resistance. The choice of U_dc_ must meet the load requirement and the grid current control requirement. Neglecting the high-order harmonics, the limits of the DC voltage can be determined by the following equations:

12 $${\text{U}}_{\text{dc}} \ge \left\{ {\begin{array}{*{20}c} {2\sqrt 2 {\text{U}}_{{ 1 {\text{abc}}}} } & {({\text{For}}\;{\text{SPWM}})} \\ {\sqrt 6 {\text{U}}_{{ 1 {\text{abc}}}} } & {({\text{For}}\;{\text{SVPWM}})} \\ \end{array} } \right.$$

13 $$\begin{array}{*{20}c} {\text{SPWM:}} & {{\text{U}}_{\text{dc}} \ge 2\sqrt 2 \left[ {\left( {{\text{U}}_{{ 1 {\text{abc}}}} + {\text{RI}}_{{ 1 {\text{abc}}}} } \right)^{2} + \left( {\upomega {\text{LI}}_{{ 1 {\text{abc}}}} } \right)^{2} } \right]^{1/2} } & {} \\ {\text{SVPWM:}} & {{\text{U}}_{\text{dc}} \ge \sqrt 6 \left[ {\left( {{\text{U}}_{{ 1 {\text{abc}}}} + {\text{RI}}_{{ 1 {\text{abc}}}} } \right)^{2} + \left( {\upomega {\text{LI}}_{{ 1 {\text{abc}}}} } \right)^{2} } \right]^{1/2} } & {} \\ \end{array}$$

where I_1abc_ is the rms value of the grid currents and R is the resistance of the filter reactor and U_1abc_ is the rms value of the grid voltages (Wang and Yin 2008). Based on Eqs. (9)–(13), the parameters used in simulation study are given in Table 2.

**Fig. 2:**
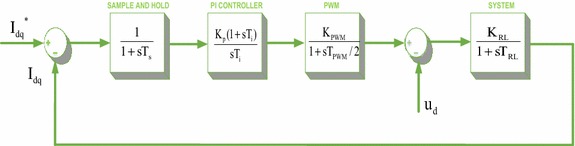
Reduced block diagram of the current control loop

The dq-axis currents must be controlled independently in the control of three-phase PWM rectifiers. The dq-axis currents are usually controlled by PI controllers with fixed paramteres. A mathematical model of the system to be controlled has been needed to obtain K_p_ and K_i_ parameters of PI controller (Blasko and Kaura 1997). The reduced block diagram is formed for the control of dq-axis currenst given in Fig. 2. The gain values of PI controller may be easily determined from this reduced block diagram.

In this reduced block diagram; T_s_ is sampling and filter delay, K_p_ is proportional gain of PI controller, T_i_ is PI controller integral time constant, T_PWM_ is time constant of the PWM block, K_PWM_ is rectifier gain K_RL_ is gain of system, T_RL_ is the system time constant. The smallest time constants in the reduced block diagram are grouped together to form a single block as a single time constant (Blasko and Kaura 1997).

14 $${\text{T}}_{\text{ei}} = {\text{T}}_{\text{s}} + {\text{T}}_{\text{PWM}} /2$$

The integral time constant of the PI controller is obtained by the following equation according to the dominant pole of the system.

15 $${\text{T}}_{\text{i}} = {\text{T}}_{\text{RL}}$$

Closed loop transfer function of the system can be obtained by using the reduced block diagram as below:

16 $${\text{H}}_{\text{ci}} = \frac{1}{{s^{2} \frac{{{\text{T}}_{\text{RL}} {\text{T}}_{\text{ei}} }}{{{\text{K}}_{\text{p}} {\text{K}}_{\text{PWM}} {\text{K}}_{\text{RL}} }} + {\text{s}}\frac{{{\text{T}}_{\text{RL}} }}{{{\text{K}}_{\text{p}} {\text{K}}_{\text{PWM}} {\text{K}}_{\text{RL}} + 1}}}}$$

To ensure 5 % overshoot, if the damping ratio is selected as (*ξ*) = $\sqrt 2 /2$, it can be obtained by the following equation:

17 $$\zeta^{2} = \frac{{{\text{T}}_{\text{RL}} }}{{ 4 {\text{K}}_{\text{p}} {\text{K}}_{\text{PWM}} {\text{K}}_{\text{RL}} }}$$

If the damping ratio is written in Eq. 16, Eq. 18 is obtained:

18 $${\text{K}}_{\text{p}} = \frac{{{\text{T}}_{\text{RL}} }}{{2{\text{T}}_{\text{ei}} {\text{K}}_{\text{PWM}} {\text{K}}_{\text{RL}} }}$$

**Fig. 3:**
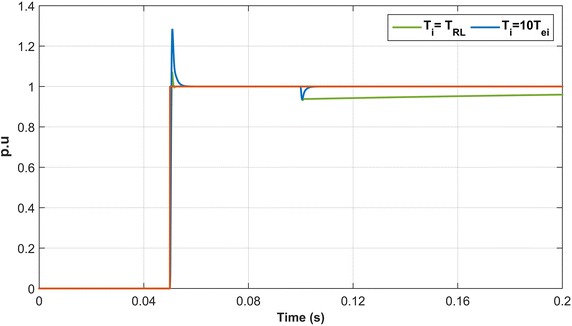
The performances of PI controllers in the current loop

The first-order transfer function can be obtained by neglecting the s^2^ term because of the very small product the term T_RL_·T_ei_.

19 $${\text{H}}_{\text{ci}} = \frac{1}{{ 1 {\text{ + sT}}_{\text{et}} }}$$

where, T_et_ = 4*T*_ei_ζ^2^, ξ = $\sqrt 2 /2$, T_et_ = 2T_ei_. The performance of the PI controller designed for the control of dq-axis currents is examined by using the reduced block diagram of the control loop (Blasko and Kaura 1997). As it is shown in Fig. 3, the time constant of the PI controller is selected as T_i_ = 10T_ei_ and T_i_ = T_RL_. Also, the responses of PI controllers are shown in Fig. 3.

**Fig. 4:**
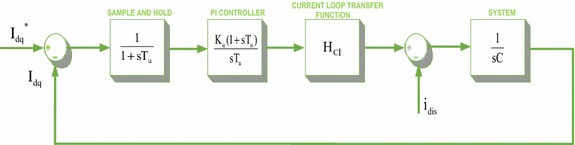
Reduced block diagram of the voltage control loop

As shown in Fig. 4, reduced block diagram is designed as in the current loop in order to control the DC voltage of PWM rectifier. In this reduced block diagram; T_du_ is the sampling and filter delay, K_u_ is PI controller proportional gain, T_u_ is PI controller integral time constant, H_ci_ is the transfer function obtained from the current loop, C is the value of the DC link capacitor.

As in the current loop, the smallest time constant of the voltage loop can be expressed as a single time constant.

20 $${\text{T}}_{\text{eu}} = {\text{T}}_{\text{du}} + {\text{T}}_{\text{ei}}$$

Gain parameters of PI controller used in control of DC voltage can be can be found by symmetric optimum method. The open-loop transfer function can be obtained as follows:

21 $${\text{H}}_{\text{cu}} = \frac{{{\text{K}}_{\text{u}} (1 + {\text{sT}}_{\text{u}} )}}{{{\text{sT}}_{\text{u}} ( 1+ {\text{sT}}{}_{\text{eu}} ) {\text{sC}}}}$$

In the symmetrical optimum method, open loop transfer function is approximately symmetrical at the crossover frequency (ω_c_). The relationship between ω_c_ and phase margin is given in Eq. (22).

22 $$\upomega_{c} = \frac{1}{{{\text{aT}}_{\text{eu}} }}\quad {\text{and}}\quad {\text{a}} = \frac{1 + \cos \upphi }{\sin \upphi }$$

The proportional gain of the PI controller can be found with the following equation for ${\text a} = \sqrt {\frac{{{\text{T}}_{\text u} }}{{{\text T}_{\text{eu}} }}}$ and a > 1.

23 $${\text{K}}_{\text{u}} = \frac{\text{C}}{{\text{aT}_{\text{eu}} }}$$

**Fig. 5:**
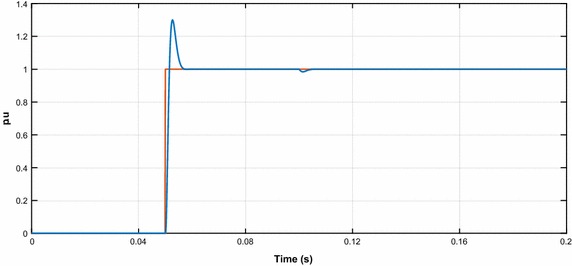
The performance of PI controller in the voltage loop

The performance of the designed PI controller for control the DC voltage of PWM rectifier is observed using a reduced block diagram of the DC voltage control loop as shown Fig. 5.

## Design of proposed controller for PWM rectifier

In recent years, NFCs which are successfully applied to many industrial applications, are based on the execution of the functions of fuzzy controller (FC) by ANN constructively (Jang et al. 1997; Zadeh 1965; Dandil and Gokbulut 2005). NFCs have non-linear structure and do not require the mathematical model of the system to be controlled. Therefore the NFCs are used widespread in the systems that are uncertain, non-linear and have parameter changes (Tuncer and Dandil 2008; Liu et al. 2003). Fuzzy rules of sugeno type FC are defined as below.

24 $${\text{R}}^{\text{j}} :\;{\text{If}}\;{\text{X}}_{1} ,{\text{A}}_{1}^{\text{j}} \ldots {\text{X}}_{\text{n}} ,A_{\text{n}}^{\text{j}} \;{\text{then}}\;{\text{y}} = {\text{f}}_{\text{j}} = {\text{a}}_{0}^{\text{j}} + {\text{a}}_{1}^{\text{j}} {\text{X}}_{1} + {\text{a}}_{2}^{\text{j}} {\text{X}}_{2} + {\text{a}}_{\text{n}}^{\text{j}} {\text{X}}_{\text{n}}$$

**Fig. 6:**
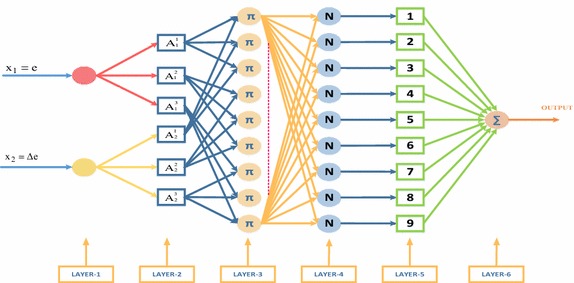
Two-inputs sugeno type NFC structure

Here, X_i_ is the input variable, y is the output variable, linguistic variables of prerequisites with A_i_^j^µ_Ai_^j^(x_i_) membership function and the *a*_*i*_^*j*^ ∊ *R* are the coefficients of linear *f*_*i*_ = (*x*_1_, *x*_2,_…, *x*_*n*_)function. Structure of NFCs which used in control algorithms is shown in Fig. 6. As seen in the Fig. 6, NFCs have two inputs, one output and six layers. Five membership functions were chosen for each input. As inputs, error (e = I_dq_*(k) − I_dq_(k) and e = V_dc_*(k) − V_dc_(k)) and the change of error (Δe = e(k) − e(k − 1)) are chosen for NFC that used in control method. ΔV_dq_ and I_d_ are obtained from the output of NFCs. At the control method, two units of NFC that shown in Fig. 6 were used totally which one is for control of DC bus voltage and the other one is for control of dq-axis currents.

In the other side, the errors and changes of reactive and active currents are given to NFC as input where NFC is used for dq-axis currents control. The amount of change of voltage that must be generated by rectifier in both dq-axis components are obtained from the outputs of NFCs that used in dq-axis current controls. Also, an external integrator that prevents integral wrapping is used for eliminate the steady state errors and limit the outputs of designed NFCs. Each neuron in the NFC’s first layer has linear activation function and transfers the input variables to its outputs. The input variables of NFC were taken as shown in Eqs. (25) and (26). The input variables of NFC are determined in the first layer. Input variables are the error and the change of error.

25 $${\text{e}}\left( {\text{k}} \right)_{1} = {\text{V}}_{\text{dc}}^{*} - {\text{V}}_{\text{dc}} \;{\text{and}}\;{\text{e}}\left( {\text{k}} \right)_{2} = {\text{I}}_{\text{dq}}^{*} - {\text{I}}_{\text{dq}}$$

26 $$\Delta {\text{e(k)}} = {\text{e(k)}} - {\text{e}}({\text{k}} - 1)$$

Membership functions are performed in the second layer where membership function is replaced by the activation function of each artificial neuro cell. Five membership functions are determined for the error and the change of error. The output of this layer is obtained as follows:

27 $${\text{net}}_{\text{j}}^{2} = - \frac{{({\text{x}}_{\text{i}} - {\text{m}}_{\text{ij}} )^{2} }}{{2({{\sigma }}_{\text{ij}} )^{2} }},\;{\text{y}}_{\text{j}}^{2} = \exp \left( {{\text{net}}_{\text{j}}^{2} } \right)$$

σ_ij_ and m_ij_, which are also input parameters, here represent the parameters of membership functions to be adapted. X_i_ represents the input of ith cell of 2nd layer. Similar to fuzzy logic controller, the third layer of NFC consists of rule base and fuzzy rules are determined in this layer.

28 $${\text{net}}_{k}^{3} = \prod_{\text{j}} {\text{w}}_{\text{jk}}^{3} {\text{x}}_{\text{j}}^{3} ,{\text{y}}_{\text{k}}^{3} = {\text{net}}_{k}^{3}$$

X_j_^3^ represents the input of jth cell of third layer. The output of the system defined by using central clarification for Mamdani fuzzy logic:

29 $${\text{net}}_{0}^{4} = \sum\limits_{\text{k}} {\text{w}}_{\text{ko}}^{4} {\text{y}}_{\text{k}}^{3} ,{\text{y}}_{0}^{4} = \frac{{\text{net}}_{0}^{4} }{\sum\nolimits_{\text{k}} {\text{y}}_{\text{k}}^{3}}$$

4th layer is called normalization layer where the accuracy of fuzzy rules are calculated. 5th layer is called as firing size of a rule. The firing degree of normalized rules are multiplied by linear f function in this layer. In order to update input and output parameters by using analog teaching method with back propagation algorithm, the squared error (E) which minimizes tracking error (e) is determined as follows (Jang et al. 1997; Dandil and Gokbulut 2005):

30 $${\text{E}} = \frac{1}{2}{\text{e}}^{2}$$

The performance index for the parameters of membership functions in PWM rectifier can be derived as follows:

31 $$\frac{{\partial {\text{E}}}}{{\partial {\text{w}}_{{{\text{k}}0}} }} = - {\text{e}}.\text{sgn} \left( {\frac{{\Delta {\text{i}}_{\text{dq}} }}{{\Delta {\text{y}}_{0}^{4} }}} \right)\frac{1}{{\mathop \sum\nolimits_{\text{k}} {\text{y}}_{\text{k}}^{3} }}w_{{{\text{k}}0}}^{4} \frac{{{\text{x}}_{\text{i}} - {\text{m}}_{\text{ij}} }}{{(\sigma_{\text{ij}} )^{2} }}{\text{y}}_{\text{j}}^{2}$$

32 $$\frac{{\partial {\text{E}}}}{{\partial {\text{w}}_{{{\text{k}}0}} }} = - {\text{e}}.\text{sgn} \left( {\frac{{\Delta {\text{V}}_{\text{dc}} }}{{\Delta {\text{y}}_{0}^{4} }}} \right)\frac{1}{{\mathop \sum\nolimits_{\text{k}} {\text{y}}_{\text{k}}^{3} }}{\text{w}}_{{{\text{k}}0}}^{4} \frac{{{\text{x}}_{\text{i}} - {\text{m}}_{\text{ij}} }}{{(\sigma_{\text{ij}} )^{2} }}{\text{y}}_{\text{j}}^{2}$$

33 $$\frac{{\partial {\text{E}}}}{{\partial \sigma_{\text{ij}} }} = - {\text{e}}.\text{sgn} \left( {\frac{{\Delta {\text{i}}_{\text{dq}} }}{{\Delta {\text{y}}_{0}^{4} }}} \right)\frac{1}{{\mathop \sum\nolimits_{\text{k}} {\text{y}}_{\text{k}}^{3} }}{\text{w}}_{{{\text{k}}0}}^{4} \frac{{({\text{x}}_{\text{i}} - {\text{m}}_{\text{ij}} )^{2} }}{{(\sigma_{\text{ij}} )^{3} }}{\text{y}}_{\text{j}}^{2}$$

34 $$\frac{{\partial {\text{E}}}}{{\partial \sigma_{\text{ij}} }} = - {\text{e}}.\text{sgn} \left( {\frac{{\Delta {\text{V}}_{\text{dc}} }}{{\Delta {\text{y}}_{0}^{4} }}} \right)\frac{1}{{\mathop \sum\nolimits_{\text{k}} {\text{y}}_{\text{k}}^{3} }}{\text{w}}_{{{\text{k}}0}}^{4} \frac{{({\text{x}}_{\text{i}} - {\text{m}}_{\text{ij}} )^{2} }}{{(\sigma_{\text{ij}} )^{3} }}{\text{y}}_{\text{j}}^{2}$$

**Fig. 7:**
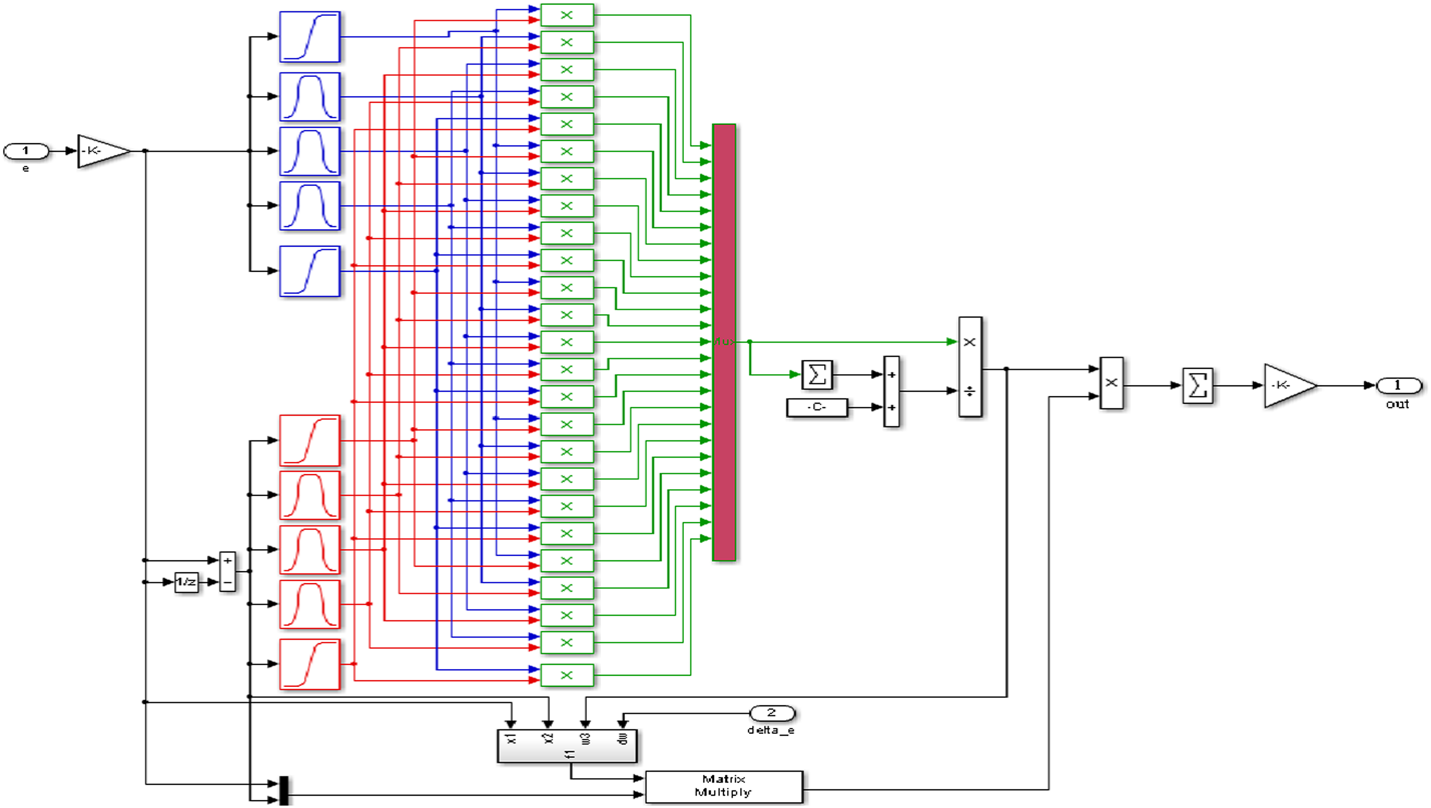
Internal structure of the NFC used in proposed control scheme

Matlab model of the NFCs used in the simulation study is shown in Fig. 7. As shown in Fig. 7, inputs of NFCs were selected as error and change in error. Five membership functions are used for each input. Membership functions were constructed to represent the input and output values.

**Fig. 8:**
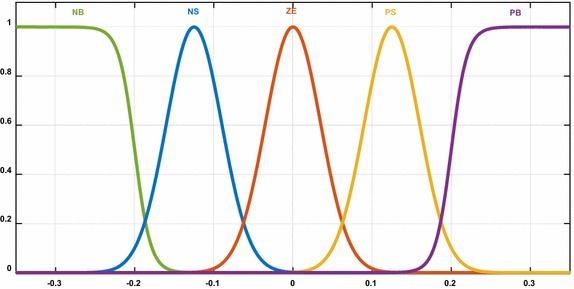
Membership functions for inputs and outputs

Figure 8 shows the fuzzy sets and corresponding bell membership function description of each signal for inputs and output. The fuzzy membership functions consist of five fuzzy sets: NB, NS, ZE, PS, PB as shown in Fig. 8.

Table 1 shows the corresponding rule table for NFCs. The top row and left column of the matrix indicate the fuzzy sets of the variables e and ∆e respectively, and the output variable ∆u are shown in the body of the matrix numerically. There may be 5 × 5 = 25 possible rules in the matrix.

**Table 1:** 5 × 5 Rule table for fuzzy inference system

∆u	∆e				
	NB	N	Z	P	PB
e					
NB	NB	NB	NB	N	Z
N	NB	N	N	N	Z
Z	NB	N	Z	P	PB
PS	Z	P	P	P	PB
PM	Z	P	PB	PB	PB

In the proposed NFC structure, precondition parameters of membership layer have been trained in the simulation model. During the simulation studies, output parameters have been trained using back-propagation learning algorithm. These parameters are adapted until the desired performance is reached.

## Simulation results

**Fig. 9:**
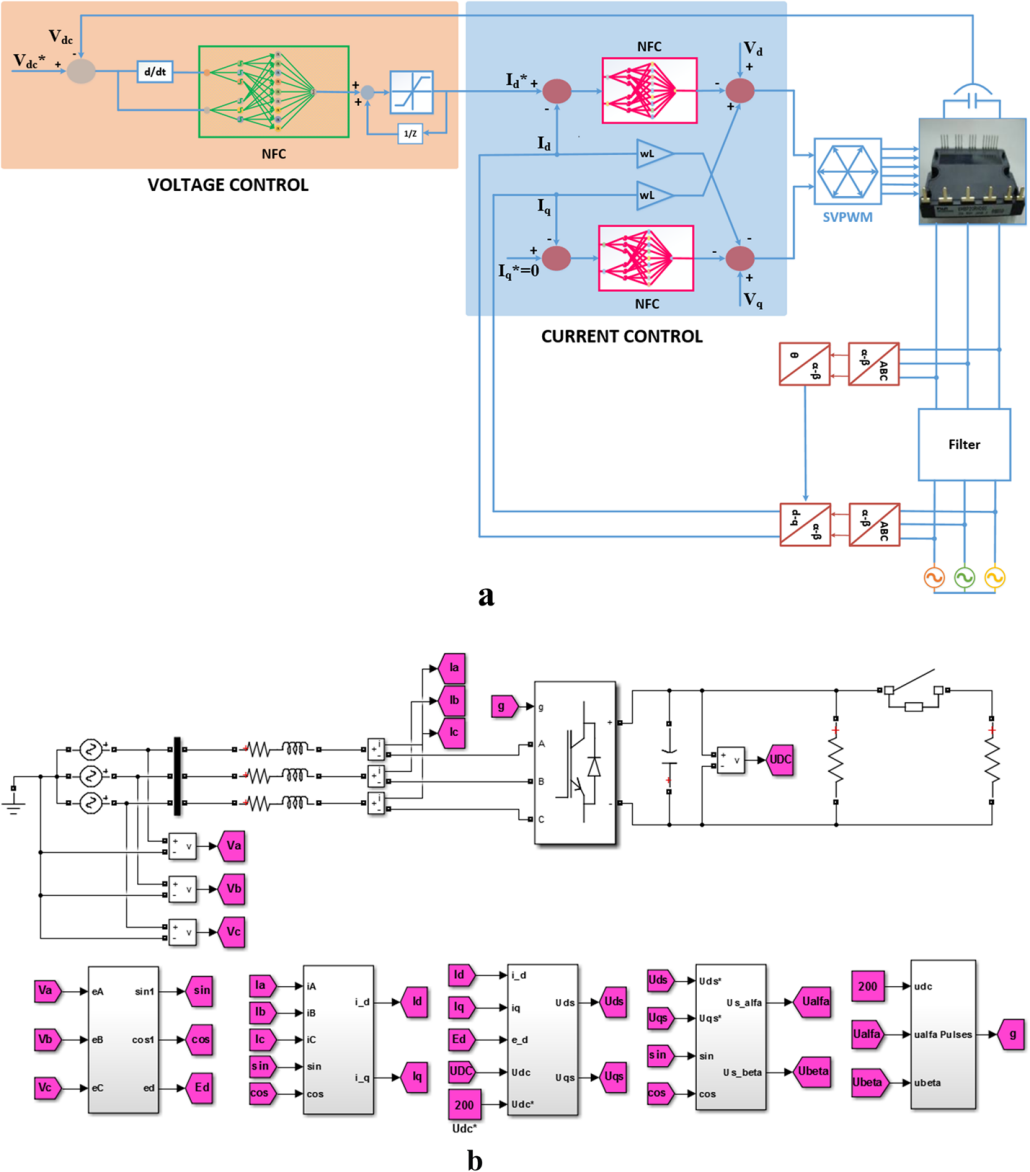
PWM rectifier model. **a** Proposed control scheme, **b** MATLAB/Simulink model of PWM rectifier

In this section, it has been carried out a number of simulation studies in order to test the dynamic performance of proposed PWM rectifier system. The proposed control scheme used in PWM rectifier system is shown in Fig. 9a. As shown in this, DC bus voltage and dq-axis currents of PWM rectifier are controlled by using NFC controllers. Also, the simulation model has designed in MATLAB/Simulink environment as shown in Fig. 9b. The transformation blocks used in the simulation model is also given in Fig. 9b.

The electrical and control parameters of PWM rectifier used in the simulation study are given in Table 2.

**Table 2:** Electrical parameters of PWM rectifier

Parameters	Value
Input grid voltage	60 V_rms_/50 Hz
Source resistance	100 mΩ
Source inductance	3.5 mH
DC bus capacitor	3.3 mF
Load resistance	20 Ω/10 Ω
Sampling time	50 µs
Switching frequency	5 kHz

**Fig. 10:**
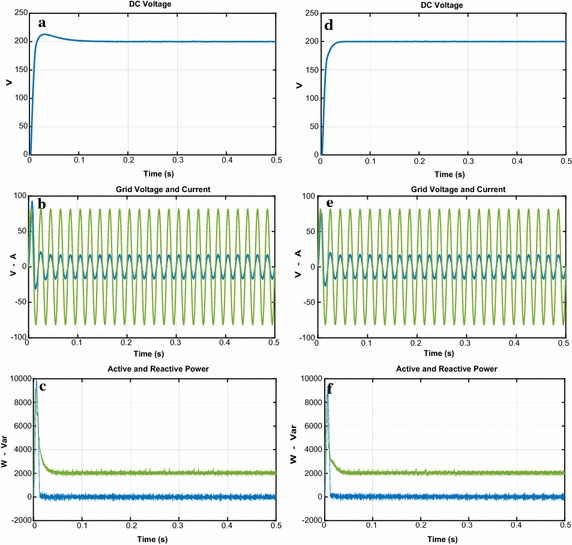
Waveforms of PWM rectifier under steady state operation for PI. **a** DC voltage, **b** grid voltage and current, **c** active and reactive power, for NFC, **d** DC voltage, **e** grid voltage and current, **f** active and reactive power

**Fig. 11:**
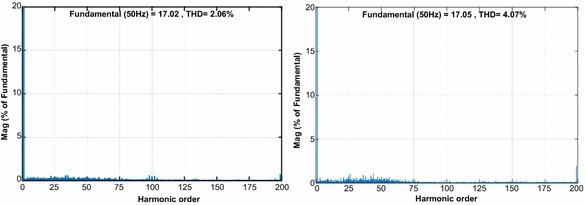
THD values of both controllers under steady state operation

The first test is realized under steady state operation as shown in Fig. 10. Reference DC voltage of PWM rectifier is set to 200 V with unity power factor. It is seen from the Fig. 10a that the PI controller reaches to reference DC voltage after 101 ms with overshoot (nearly 7.15 %). Moreover, the input currents have nearly sinusoidal waveforms and in phase with grid voltage thus a unity power factor is nearly 0.99 and THD value is 4.07 %. As it can be seen from the Fig. 10d, the proposed controller reaches to reference DC voltage after 32 ms without overshoot. Also, the proposed control scheme has power factor of 1 and THD of 2.06 %. It is noticed that supply current in phase with grid voltage and so unity power factor is obtained. The waveforms related to active and reactive power are shown in Fig. 10c–f. It can be seen that active power close to new reference value and reactive power is zero because of the unity power factor operation. It can be seen that the performance of NFC is much better than PI controller in terms of settling time, maximum peak overshoot. Moreover, as shown in Fig. 11, THD levels of gird current for PI and controller and NFC under steady state operation are 4.07 and 2.06 %, respectively. These THD levels are smaller than specific limit that is mentioned IEEE 519-1992 standard.

**Fig. 12:**
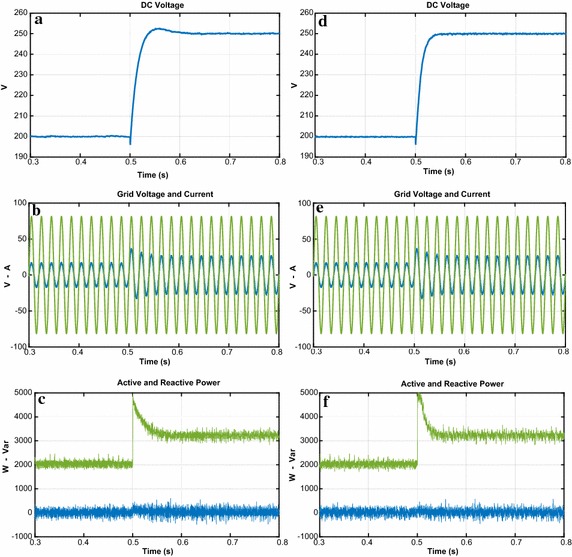
Waveforms of PWM rectifier under transient state operation for PI. **a** DC voltage, **b** grid voltage and current, **c** active and reactive power, for NFC, **d** DC voltage, **e** grid voltage and current, **f** active and reactive power

**Fig. 13:**
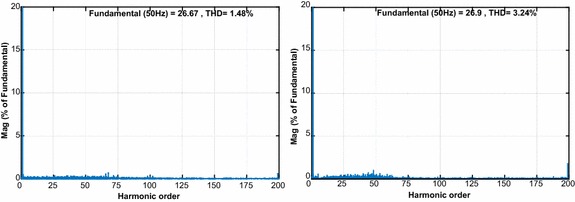
THD values of both controllers under transient state operation

The second test is realized in order to demonstrate the dynamic performance of proposed control scheme and PI controller under step response. Figure 12 shows that the reference DC voltage steps up to 200 V from 250 V at 0.5 s. When applied the step DC voltage, PI controller response reaches the reference DC voltage at 0.6 s with overshoot while the proposed controller follows the reference DC voltage after 0.0358 s without overshoot and steady state error. The line currents given in Fig. 12b–e have clearly sinusoidal waveforms and in phase with grid voltage. Due to its good performance, the output active power of proposed controller reaches to its desired value faster than PI controller. Moreover, the reactive power response of proposed controller is rapidly regulated to zero. Figure 12 is clearly specied that reactive power control and active power is performed more effective with proposed controller. As shown in Fig. 13, THD levels of gird current for PI controller and NFC under transient state operation are 3.24 % and 1.48 %, respectively.

**Fig. 14:**
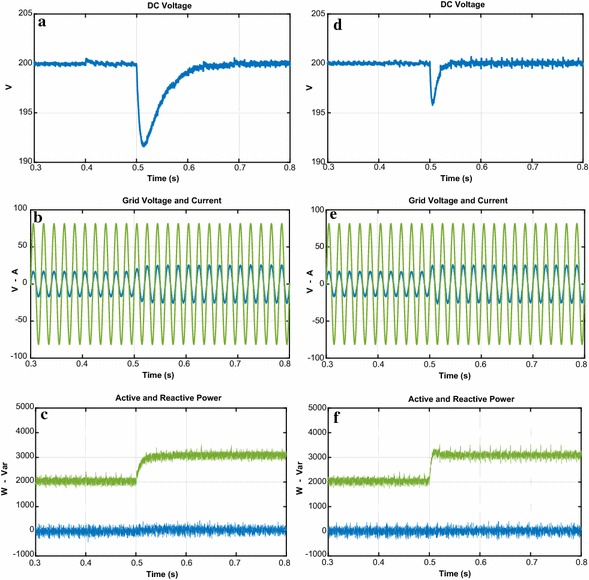
Waveforms of PWM rectifier in load change condition for PI. **a** DC voltage, **b** grid voltage and current, **c** active and reactive power, for NFC, **d** DC voltage, **e** grid voltage and current, **f** active and reactive power

The third test is realized in order to indicate the performance of both controllers against the load change. The DC bus voltage responses with load at 0.3 s are given in Fig. 14a–d. When the load is applied, there is sudden dip in DC voltage. The DC voltage response of proposed controller falls from 200 to 196.5 V and it takes 0.021 s to reach the reference DC voltage whereas the DC voltage response of PI controller dips from 200 to 191 V and reaches after 0.125 s. Moreover, the reactive power responses of both controllers are shown in Fig. 14c–f. It can be seen from these figures that reactive power is zero despite the load change.

**Fig. 15:**
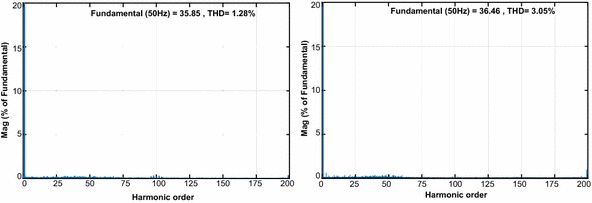
THD values of both controllers under load change condition

As shown in Fig. 15, THD values of both controllers under load change condition were analysed in order to better verify the success of the proposed controller in terms of THD and power quality. THD values of both controllers under load change condition are 1.28 % and 3.05 %, respectively. The study demonstrates that the proposed control scheme based PWM rectifier improves THD and power quality.

**Fig. 16:**
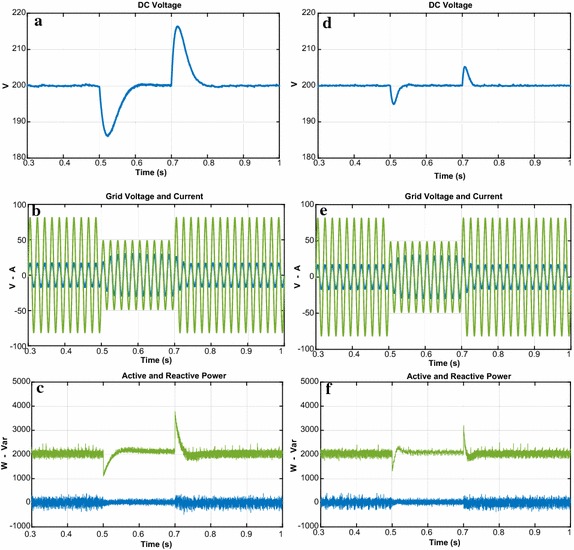
Waveforms of PWM rectifier in voltage sag condition for PI. **a** DC voltage, **b** grid voltage and current, **c** active and reactive power, for NFC, **d** DC voltage, **e** grid voltage and current, **f** active and reactive power

**Fig. 17:**
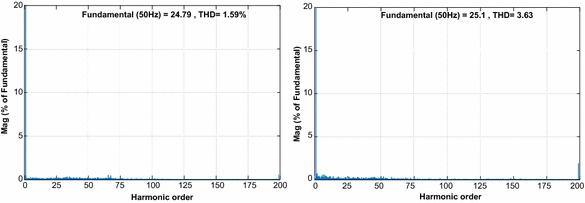
THD values of both controllers under voltage sag condition

The fourth test is carried out to demonstrate the response of both controllers under voltage sag condition as shown in Fig. 16. In this test, the magnitude of grid voltage changes from 100 to 70 % at 0.5 s, and then changes back from 70 to 100 % at 0.7 s. When the voltage sag happens at t = 0.5 s, the enlarged DC voltage obtained from PI controller falls nearly 187 V and reaches reference DC voltage at 0.8 s whereas proposed controller falls 198 V and reaches reference DC voltage at 0.73 s. As clearly seen in Fig. 16d, although voltage sag at input stage is occurred, DC bus voltage response obtained from proposed controller is more durable than PI controller. Active power is nearly constant and reactive power is zero because of good performance of controllers. The line currents given in Fig. 16b–e have clearly sinusoidal waveforms and in phase with grid voltage. As shown in Fig. 17, THD values of both controllers under voltage sag condition were analysed and THD levels of NFC and PI controller are 1.59 % and 3.63 %, respectively.

**Fig. 18:**
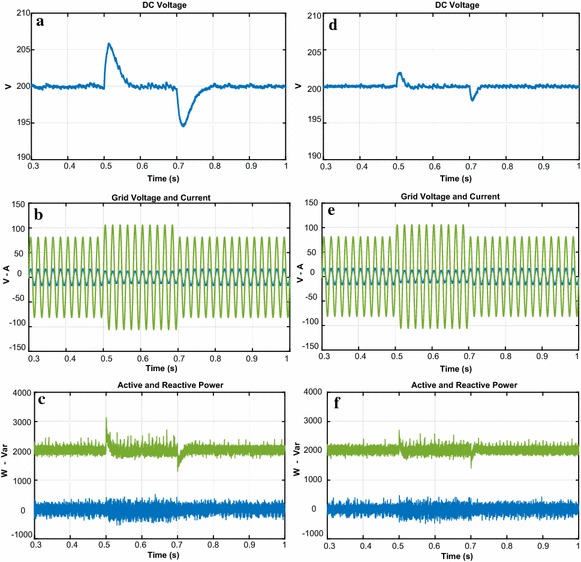
Waveforms of PWM rectifier in voltage swell condition for PI. **a** DC voltage, **b** grid voltage and current, **c** active and reactive power, for NFC, **d** DC voltage, **e** grid voltage and current, **f** active and reactive power

**Fig. 19:**
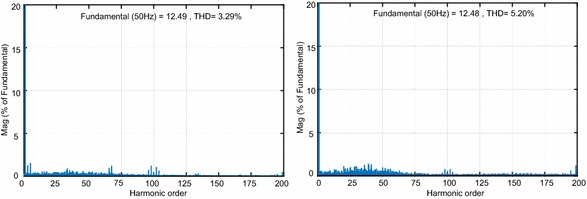
THD values of both controllers under voltage swell condition

The last test is realized in order to indicate the effectiveness of both controllers against voltage swell condition. Figure 18 shows the simulation results when the grid voltages occur 30 % of three-phase voltage swells at t = 0.5 to 0.7 s. According to Fig. 18a–d, when the voltage swell occurs, NFC controller rises 201.56 V and reaches to reference DC bus voltage at 0.72 s whereas DC voltage obtained from PI controller rises 206 V and reaches reference DC voltage at 0.8 s. Figure 18c–f depict that after change in the grid voltages, active power is rapidly regulated at the desired value without steady-state error. NFC has much better reference tracking after voltage swell have happened. Also, the line currents in given Fig. 18b–e have clearly sinusoidal waveforms and in phase with grid voltage. Total harmonic distortion levels of of the both controllers under voltage swell condition are shown in Fig. 19. As clearly seen in Fig. 19, the proposed control scheme based PWM rectifier system has better performance than PI controller in terms of THD.

## Conclusion

In this paper, a robust control scheme has proposed and developed using neuro-fuzzy controller for PWM rectifier. The emphasis was on the analysis, design and implementation of the proposed control scheme in MATLAB/Simulink environment. First, parameters of PI controller, which needs the mathematical model of the system to be controlled, are designed according to reduced block diagram. Neuro-fuzzy controller structure for control of DC voltage and dq-axis currents of PWM rectifier has developed via MATLAB/Simulink blocks. Designed control scheme is applied to the PWM rectifier and neuro-fuzzy structure is trained until obtaining the desired results. After designing of both controllers, simulation tests are realized for both controllers at the same conditions. According to simulation results, proposed control scheme gives more superior performance than PI controller with respect to rise time, settling time, overshoot, THD and PF in all test conditions. Moreover, proposed control scheme provides the desired reactive power exact and fast within own rated power limits even in the whole operating conditions.
